# Neurophysiological evidence of motor preparation in inner speech and the effect of content predictability

**DOI:** 10.1093/cercor/bhad389

**Published:** 2023-11-06

**Authors:** Lawrence K-h Chung, Bradley N Jack, Oren Griffiths, Daniel Pearson, David Luque, Anthony W F Harris, Kevin M Spencer, Mike E Le Pelley, Suzanne H-w So, Thomas J Whitford

**Affiliations:** School of Psychology, University of New South Wales (UNSW Sydney), Mathews Building, Library Walk, Kensington NSW 2052, Australia; Department of Psychology, The Chinese University of Hong Kong, 3/F Sino Building, Chung Chi Road, Shatin, New Territories, Hong Kong SAR, China; Research School of Psychology, Australian National University, Building 39, Science Road, Canberra ACT 2601, Australia; School of Psychological Sciences, University of Newcastle, Behavioural Sciences Building, University Drive, Callaghan NSW 2308, Australia; School of Psychology, University of Sydney, Griffith Taylor Building, Manning Road, Camperdown NSW 2006, Australia; Department of Basic Psychology and Speech Therapy, University of Malaga, Faculty of Psychology, Dr Ortiz Ramos Street, 29010 Malaga, Spain; Westmead Clinical School, University of Sydney, 176 Hawkesbury Road, Westmead NSW 2145, Australia; Brain Dynamics Centre, Westmead Institute for Medical Research, 176 Hawkesbury Road, Westmead NSW 2145, Australia; Research Service, Veterans Affairs Boston Healthcare System, and Department of Psychiatry, Harvard Medical School, 150 South Huntington Avenue, Boston MA 02130, United States; School of Psychology, University of New South Wales (UNSW Sydney), Mathews Building, Library Walk, Kensington NSW 2052, Australia; Department of Psychology, The Chinese University of Hong Kong, 3/F Sino Building, Chung Chi Road, Shatin, New Territories, Hong Kong SAR, China; School of Psychology, University of New South Wales (UNSW Sydney), Mathews Building, Library Walk, Kensington NSW 2052, Australia; Brain Dynamics Centre, Westmead Institute for Medical Research, 176 Hawkesbury Road, Westmead NSW 2145, Australia

**Keywords:** contingent negative variation, inner speech, internal forward model, readiness potential, stimulus predictability

## Abstract

Self-generated overt actions are preceded by a slow negativity as measured by electroencephalogram, which has been associated with motor preparation. Recent studies have shown that this neural activity is modulated by the predictability of action outcomes. It is unclear whether inner speech is also preceded by a motor-related negativity and influenced by the same factor. In three experiments, we compared the contingent negative variation elicited in a cue paradigm in an active vs. passive condition. In Experiment 1, participants produced an inner phoneme, at which an audible phoneme whose identity was unpredictable was concurrently presented. We found that while passive listening elicited a late contingent negative variation, inner speech production generated a more negative late contingent negative variation. In Experiment 2, the same pattern of results was found when participants were instead asked to overtly vocalize the phoneme. In Experiment 3, the identity of the audible phoneme was made predictable by establishing probabilistic expectations. We observed a smaller late contingent negative variation in the inner speech condition when the identity of the audible phoneme was predictable, but not in the passive condition. These findings suggest that inner speech is associated with motor preparatory activity that may also represent the predicted action-effects of covert actions.

## Introduction

A slow negative wave as measured by electroencephalogram (EEG) can be observed in the few seconds leading up to the execution of a voluntary movement. This premovement signature is thought to reflect brain activity that is involved in planning and preparing for an impending movement (for a review, see Brunia et al. 2012). Depending on the experimental context, two types of slow cortical potentials can be elicited: the readiness potential (RP) and the contingent negative variation (CNV).

The RP emerges when an individual is preparing to make a self-paced movement, such as pressing a button (Deecke et al. 1969; Shibasaki and Hallett 2006). It is divided into two components: an early component that begins around 2000 ms and a late component that starts around 500 ms preceding a movement (Deecke et al. 1976; Shibasaki et al. 1980; Shibasaki and Hallett 2006). The early and late RP are thought to be involved in the selection of motor strategies and the specification of movement parameters, such as force, velocity, and timing, respectively (Deecke et al. 1976; Brunia et al. 2012). The RP has been associated with cortical and subcortical sources originating in the basal ganglia-supplementary motor area (SMA)-medial premotor structures and spreading to more lateral cerebellum-motor cortex structures prior to movement onset (Lang et al. 1991; Pedersen et al. 1998; Ball et al. 1999; Yazawa et al. 2000; Ikeda and Shibasaki 2003; Fried et al. 2011).

The CNV is a similar negative-going slow wave that occurs when an individual is asked to make a quick response to an imperative stimulus (S2) following a warning stimulus (S1) (Walter et al. 1964; Rohrbaugh et al. 1976; Brunia and van Boxtel 2001). While the early CNV is related to orientation toward S1, the late CNV resembles the late RP as a movement-related component when a motor response is cued (Rohrbaugh et al. 1976; Gaillard 1977; Rohrbaugh and Gaillard 1983; Kotani et al. 2011) and is influenced by the same movement parameters as the late RP (Low and McSherry 1968; Rohrbaugh et al. 1976; van Boxtel et al. 1993). Indeed, brain imaging studies have implicated shared neural generators between self-initiated and stimulus-driven movements (Jahanshahi et al. 1995; Jenkins et al. 2000; Cunnington et al. 2002; Nagai et al. 2004). However, the late CNV also comprises nonmotoric components, such as anticipatory attention and sensory preparatory activities, that have been demonstrated to exhibit distinct topographical distributions and receive contributions from more posterior cortical areas compared with the motoric components (Leynes et al. 1998; Gomez et al. 2001, 2003, 2004, 2007), suggesting that it is not completely identical to the late RP (Donchin et al. 1972; Brunia and Damen 1988; van Boxtel and Brunia 1994a, 1994b; Ikeda et al. 1997; Cui et al. 2000).

The RP and CNV have been shown to precede different types of overt actions, including limb movements, orofacial movements, and speech (Deecke et al. 1969; Michalewski et al. 1977; Deecke et al. 1986; Wohlert 1993; McArdle et al. 2009; Vanhoutte et al. 2015). Less is known, however, about the extent to which similar patterns are also observed for purely internal “mental actions.” In particular, it is unclear whether inner speech is also preceded by a motor-related slow negative wave. Inner speech—the silent production of words in one’s mind—is a ubiquitous aspect of human cognition and is thought to play an important role in various mental processes, such as problem-solving, decision-making, and self-regulation (Perrone-Bertolotti et al. 2014; Alderson-Day and Fernyhough 2015; Alderson-Day et al. 2018). An influential account suggests that inner speech may be conceived as a special case of overt speech—“a kind of action” without articulatory movements—that constitutes our most complex motor act (Hughlings Jackson 1958; Feinberg 1978; Jones and Fernyhough 2007; Oppenheim and Dell 2010). Evidence for this idea comes from studies showing that similar brain regions to overt speech are activated during the production of inner speech, including the SMA and perceptual and language areas (McGuire et al. 1996; Zatorre et al. 1996; Palmer et al. 2001; Aleman et al. 2005; Shuster and Lemieux 2005). Recent neurophysiological studies have also demonstrated that inner speech, like overt speech, modifies the brain’s electrophysiological response to external stimuli as indexed by poststimulus event-related potentials (ERPs) (Tian and Poeppel 2010, 2013, 2015; Ylinen et al. 2015; Whitford et al. 2017; Tian et al. 2018; Jack et al. 2019). For example, when an inner phoneme was produced, the amplitude of the N1 component evoked by an audible phoneme was smaller if the two phonemes were matched in content and timing (Whitford et al. 2017; Jack et al. 2019). Yet, whether inner speech is associated with a prestimulus ERP remains an open question.

Several studies have provided preliminary support for a motor-related CNV and RP preceding imagined motor movements (Beisteiner et al. 1995; Cunnington et al. 1996, 1997; Jankelowitz and Colebatch 2002; do Nascimento et al. 2006; Niazi et al. 2013; Park et al. 2022). For example, Park et al. (2022) asked participants to complete the Libet clock task, which involved reporting the timing of a button press in relation to the position of a rotating clock hand on a clock face. In addition to a motor execution condition, participants were also asked to imagine pressing the button without any physical movement, or to visualize the clock hand stopping. The results showed that RP was present not only before actual button presses, but also before imagined button presses and imagined visual motion, although the RPs were of smaller amplitude in these last two cases. Thus, the presence of RP before motor and visual imagery was demonstrated. In the domain of speech imagery, a recent study asked participants to press a button and simultaneously imagine vocalizing the syllable “ah” every few seconds (Pinheiro et al. 2020b). In a separate condition, the button press was always followed by the same audible speech sound. It was found that the early and late RP in both conditions were more negative, relative to a control condition where the button press was not followed by any stimulus. These findings are consistent with the idea that the production of inner speech has an impact on the RP. However, the overt action (i.e. a button press) and its related muscle movement occurred concurrently with inner speech, which limits the interpretation of an inner-speech-*specific* RP. In short, accumulating evidence supports the notion that overt and covert actions may both elicit motor preparation programs (Jeannerod and Decety 1995; Tian and Poeppel 2012), but their role in inner speech remains open to debate.

Finally, a growing body of literature has indicated that preparatory neural activity not only reflects motor preparation, but may also instantiate expected sensory consequences of the motor action (Ford et al. 2014; Reznik et al. 2018; Vercillo et al. 2018; Wen et al. 2018; Pinheiro et al. 2020b). According to the internal forward model, a predictive signal coined an “efference copy” is sent to the sensory areas based on a copy of the motor command from the motor areas during a voluntary movement to anticipate, and subsequently modulate, the resultant sensory consequences, which serves to discern self-generated sensations from externally generated input (Wolpert et al. 1995; Wolpert and Miall 1996; Wolpert et al. 2011). Hence, the efference copy is thought to be embedded in the premovement slow wave (Ford et al. 2014). Accordingly, recent studies have reported a larger RP amplitude in actions that were followed by predictable sensory effects (Reznik et al. 2018; Vercillo et al. 2018; Wen et al. 2018), even when the effect was imagined (Pinheiro et al. 2020b), compared with actions without any effects. A body of research has also demonstrated a link between premovement activity and specific sensory feedback by showing that presenting auditory probes during overt speech preparation resulted in reduced amplitude of ERPs compared with nonspeaking conditions (Daliri and Max 2016; Max and Daliri 2019). This raises the possibility that stimulus predictability may also affect the amplitude of preparatory motor activity in inner speech.

The aims of the present study are twofold. First, we aimed to establish the existence of a motor-related slow wave to inner speech in Experiment 1. We employed a ticker-tape-style cue paradigm as detailed in Whitford et al. (2017) to precisely time the onset of an inner phoneme while EEG was simultaneously recorded. Based on the cueing nature of the experimental paradigm (in which participants were externally cued to produce inner speech at a particular moment), the slow negative wave is denoted as a CNV, rather than an RP, which typically involves self-initiated movements (Brunia et al. 2012), though similar neural processes may underlie these components (Schurger et al. 2021). To control for the contributions of nonmotoric components to the CNV, we compared the late CNV in the inner speech condition with a passive condition in which no inner speech was produced. We hypothesized a larger late CNV amplitude in the inner speech condition than the passive condition. In Experiment 2, we attempted to provide a point of reference by conducting an identical experiment to Experiment 1, except that participants were asked to overtly (as opposed to covertly) vocalize the phoneme. We hypothesized a similar pattern of results to Experiment 1, in that a larger late CNV amplitude in the overt speech condition than the passive condition would be observed, which would support the idea of a functional similarity between inner and overt speech, i.e. that inner speech is a special case of overt speech (Feinberg 1978; Jones and Fernyhough 2007). In Experiment 3, we explored the influence of stimulus predictability on the late CNV of inner speech by manipulating the conditional probability of an audible phoneme presented simultaneously at the onset of an inner phoneme, such that prior expectations of the stimulus identity could be formed during inner speech preparation. We hypothesized an effect of stimulus predictability on the late CNV for the inner speech conditions where action-effect contingency could be established but not for the passive conditions.

## Experiment 1

### Materials and methods

#### Participants

Fifty participants enrolled in a first-year Psychology course at UNSW Sydney were recruited in exchange for course credit. All participants provided informed written consent. Nine participants were excluded from the analysis for generating <50% of usable epochs in one or more conditions. The final sample consisted of 41 participants, of whom 21 were female, with a mean age of 23.59 (*SD* = 7.38) years. All participants self-reported to have normal or corrected-to-normal vision and hearing. All reported experiments were approved by UNSW Sydney Human Research Ethics Advisory Panel and conducted in accordance with the Declaration of Helsinki.

#### Apparatus, stimuli, and procedure

**Fig. 1:**
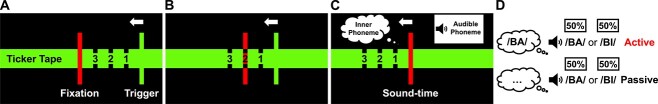
**Experiment 1 protocol schematic. (A)** Participants were asked to fixate their eyes on the red fixation line in the middle of the screen. **(B)** After a delay of 1–2 s, the green trigger line began moving leftwards smoothly across the screen from the far right-hand side of the screen at a speed of 6.5°/s. **(C)** After 3.75 s, the trigger line overlapped with the fixation line—A moment dubbed the “sound-time.” At this precise moment, two events took place simultaneously. **(D)** In the *active condition*, participants were instructed to silently produce a predefined phoneme (either /BA/ or /BI/) in their heads. In the *passive condition*, participants were instructed to passively listen to the audible phoneme. At the same time, an audible phoneme (either /BA/ or /BI/), produced by a male speaker with a loudness of 70 dB SPL, was delivered to the participants’ headphones. Each audible phoneme (/BA/ and /BI/) was presented on 50% of trials within each trial block in a randomized order. After the sound-time, the trigger line continued to move past the fixation line for an additional 1 s and stopped. The trial was then completed.

The experimental setup has been described in detail previously (Whitford et al. 2017). Participants were seated in a quiet, dimly-lit room, ~60 cm in front of a computer monitor (BenQ XL2420T, 1920 × 1080 pixels, 144 Hz) and were fitted with a pair of headphones (AKG K77 Perception). On each trial, participants watched a countdown animation: It started with a thick green horizontal line (i.e. the ticker tape) in the middle of the screen, a red vertical line (i.e. the fixation line) at the center of the screen, and a green vertical line (i.e. the trigger line) on the far right-hand side of the screen, as illustrated in Figure 1A. Participants were instructed to focus their gaze on the fixation line (which remained stationary) for the duration of a trial. The ticker tape was marked with labels “3,” “2,” and “1” that past the fixation line 3 s, 2 s, and 1 s prior to the trigger line. With a constant velocity of 6.5°/s, the trigger line moved across the screen toward the fixation line (see Fig. 1B), such that after 3.75 s the two lines intersected; this point was termed the sound-time. At this moment, an *audible phoneme* /BA/ or /BI/ was delivered to participants’ headphones; this was produced by a male speaker, with a duration of 200 ms and loudness of 70 dB SPL (see Fig. 1C). Given that previous studies have reported distinct neural correlates and brain activation of voice gender processing (Sokhi et al. 2005; Zäske et al. 2009; Latinus and Taylor 2012; Weston et al. 2015), a single (male) voice sample was consistently used across all the experiments. Each audible phoneme (/BA/ and /BI/) was presented on 50% of trials within each trial block in a randomized order. Thus, the content of the audible phoneme was unpredictable for any given trial prior to the sound-time. The trigger line then continued to move past the fixation line for an additional 1 s, after which it stopped.

The experiment consisted of 6 trial blocks, with 60 trials in each block. On two-thirds of the blocks, the participants were asked to silently produce a particular *inner phoneme* (either /BA/ or /BI/) in their heads at the exact moment the fixation line intersected the trigger line (i.e. at the sound-time)—the *Active* condition. They were asked to always produce the same inner phoneme within a given block, and when doing so imagine themselves moving their articulator organs (i.e. mouth, tongue, larynx, etc.) and vocalizing the inner phoneme, but without actually making any movements. On one-third of the blocks, the participants were asked to simply listen to the audible phoneme and not to imagine anything in particular—the *Passive* condition (see Fig. 1D). The order of the trial blocks was randomized. After each trial, participants were asked to rate their subjective performance in successfully completing the task with a 5-point Likert scale, which ranged from 1 (“Not at all successful”) to 5 (“Completely successful”), using the computer keypad. These ratings were used to exclude trials in both conditions in which participants failed to perform the task (i.e. failure in imagining the instructed inner phoneme at the sound-time in the Active condition or in not imagining any inner phoneme in the Passive condition). Stimulus presentation was controlled by MATLAB (MathWorks, Natick, Massachusetts) using the Psychophysics Toolbox extensions (Brainard 1997; Kleiner et al. 2007).

#### E‌EG acquisition

EEG was recorded with a BioSemi ActiveTwo system from 64 Ag/AgCl active electrodes placed according to the extended 10–20 system (FP1, FPz, FP2, AF7, AF3, AFz, AF4, AF8, F7, F5, F3, F1, Fz, F2, F4, F6, F8, FT7, FC5, FC3, FC1, FCz, FC2, FC4, FC6, FT8, T7, C5, C3, C1, Cz, C2, C4, C6, T8, TP7, CP5, CP3, CP1, CPz, CP2, CP4, CP6, TP8, P9, P7, P5, P3, P1, Pz, P2, P4, P6, P8, P10, PO7, PO3, POz, PO4, PO8, O1, Oz, O2, Iz), with an online reference of CMS and DRL sites. A vertical electro-oculogram (EOG) was recorded by placing an electrode below the left eye, and subtracting its activity from that of electrode FP1; a horizontal EOG was recorded by placing an electrode on the outer canthus of each eye. We also placed an electrode on the tip of the nose, on the left and right mastoid, and on the masseter muscle to detect jaw movements. The sampling rate was 2048 Hz.

#### E‌EG processing and analysis

The data preprocessing was performed in BrainVision Analyzer (Brain Products GmbH, Munich, Germany). The EEG data were re-referenced offline to the average of the mastoid electrodes. Data were first notch filtered at 50 Hz to remove mains artifact, and then band-pass filtered from 0.1 to 30 Hz using a phase-shift free Butterworth filter (12 dB/Oct slope). The present study reported on the prestimulus ERPs only (i.e. prior to sound onset), and as such a distinction of content congruency between the inner and audible phonemes could not be applied. For poststimulus ERPs results (and specifically comparisons of match and mismatch trials), see Whitford et al. (2017). Epochs were extracted from −2500 to 100 ms (i.e. 2600 ms epochs) relative to the sound onset, and were baseline-corrected to their mean voltage from −2500 to −2000 ms (Vanhoutte et al. 2015). All epochs were corrected for eye-movement artifacts using the technique described in Gratton et al. (1983) and Miller et al. (1988). Any epochs with signals exceeding peak-to-peak amplitudes of 200 μV at any EEG channel was excluded. To further ensure data quality, any epochs in which participants rated their performance of the trial as less than 3 out of 5 were excluded. The remaining usable epochs were included in the analysis. There was an average of 200.56 (*SD* = 25.95) usable epochs in the *Active* condition (of a max. possible 240 epochs), and 106.78 (*SD* = 12.63) in the *Passive* condition (of a max. possible 120 epochs).

The ticker-tape design of the present study differs from a traditional S1-S2 design of CNV studies (e.g. Michalewski et al. 1977; Vanhoutte et al. 2015) in that there is no clear onset of S1. Therefore, we opted to focus the analysis on the late CNV, which typically can be observed prior to the onset of S2 (i.e. sound-time). The dependent variable was the mean amplitude of the late CNV in the 500 ms time-window preceding stimulus onset. The time-window was selected after visual inspection of the ERPs and voltage maps, and to be consistent with previous studies that examined prestimulus slow negative waves in preparation of speech (Galgano and Froud 2008; Vanhoutte et al. 2015; Ning et al. 2017) and finger movements (Vercillo et al. 2018; Wen et al. 2018; Pinheiro et al. 2020b). Average voltage within this time-window was the dependent variable.

#### Statistical analysis

**Fig. 2:**
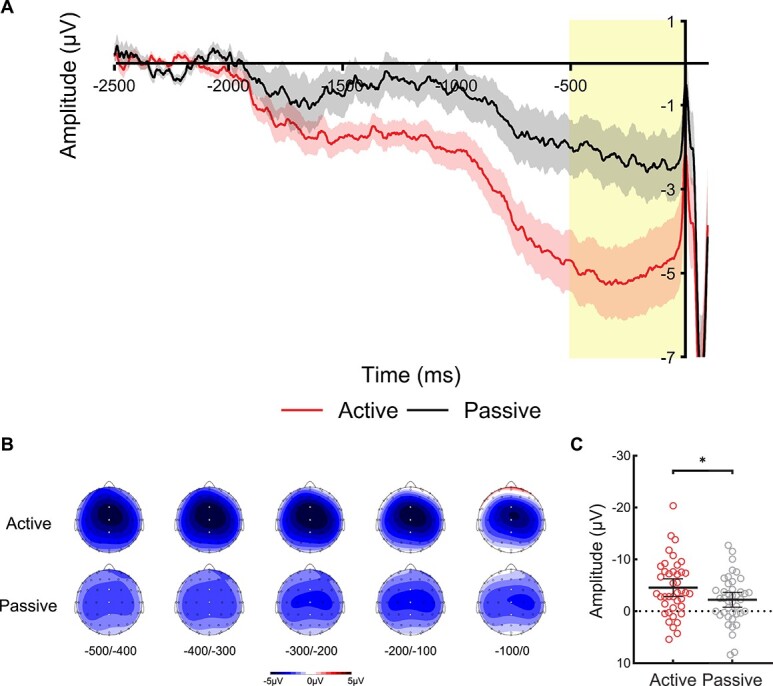
**CNV analysis of Experiment 1. (A)** Waveforms show the ERPs during the prestimulus period at electrode Cz in the active condition (red/bottom line) and passive condition (black/top line), and the shaded area represents the standard error of the mean. The yellow bar shows the late CNV time-window (−500 to 0 ms). The waveforms are shown collapsed across trials with audible phoneme /BA/ and /BI/ at stimulus onset (0 ms), and the waveform for the active condition is shown collapsed across inner phoneme /BA/ and /BI/ during the inner speech task. **(B)** Voltage maps are plotted separately for each condition showing the distribution of voltages over the scalp during the late CNV time-window for successive 100 ms time-windows from −500 ms until stimulus onset at 0 ms; white dots illustrate the electrodes FCz, Cz, and CPz used in the analysis. **(C)** Scatterplots show the mean amplitude of the late CNV time-window for the active and passive conditions averaged across electrodes FCz, Cz, and CPz. Each point represents one participant’s mean amplitude averaged across trials. These individual mean amplitudes were approximately normally distributed with no clear outliers. The horizontal bars represent the mean, and the error bars represent the 95% confidence interval. A significant contrast (*P* < 0.05) is indicated with an asterisk.

Data were analyzed using SPSS version 26.0. First, to test for the existence of a late CNV waveform, one-sample *t*-tests (two-tailed) were performed for the *Active* and *Passive* conditions to test whether the mean amplitude was significantly more negative than zero in each of these conditions (Park et al. 2022). Next, to compare the amplitude of the late CNV between the *Active* and *Passive* conditions, a paired-sample *t*-test (two-tailed) was performed with the mean amplitude of the late CNV. To improve the reliability of the results, all analyses were conducted collapsing across the midline electrodes FCz, Cz, and CPz, as these were the electrodes at which the late CNV was maximal based on visual inspection (see Fig. 2B, voltage maps), and to be consistent with previous studies on preparatory motor activity during overt speech, which typically focused on midline electrodes (Wohlert 1993; McArdle et al. 2009; Vanhoutte et al. 2015; Ning et al. 2017). Given the hypothesis-driven nature of our study and the limited number of specific comparisons, correction for multiple tests was not applied in the analyses. The comparisons were preplanned based on prior research that is relevant to our research questions.

### Results

Figure 2A shows the ERPs during the prestimulus period at electrode Cz. Figure 2B shows the voltage maps during the late CNV time-window for successive 100 ms time-windows from 500 ms before stimulus onset. Figure 2C shows a scatterplot of the mean amplitude of the late CNV time-window averaged across electrodes FCz, Cz, and CPz for each condition. These individual mean amplitudes were approximately normally distributed with no clear outliers. One-sample *t*-tests revealed that the late CNV amplitude was significantly greater than the baseline in both the *Active* condition, *t*(40) = 5.44, *P* < 0.001, *d* = 0.85, 95% CI = (−6.278, −2.878), and in the *Passive* condition, *t*(40) = 3.16, *P* = 0.003, *d* = 0.49, 95% CI = (−3.674, −0.809). This indicates that a late CNV waveform was elicited in both conditions, which is maximal over the medial region and has a bilateral topographic distribution (see Fig. 2B, voltage maps). Moreover, a paired-sample *t*-test found that the late CNV amplitude in the *Active* condition was significantly larger than the *Passive* condition, *t*(40) = 3.37, *P* = 0.002, *d* = 0.53, 95% CI = (−3.739, −0.934). This indicates that the preparation to produce inner speech led to a significantly more negative late CNV than passive listening.

An exploratory analysis revealed no significant difference in the late CNV between match (e.g. imagine /BA/ and hear /BA/ at the sound-time) and mismatch trials (e.g. imagine /BA/ and hear /BI/ at the sound-time), *t*(40) = 0.42, *P* = 0.675, *d* = 0.07, 95% CI = (−0.688, 1.053).

In Experiment 2, we sought to provide a control check of the pattern of results by conducting an identical experiment to Experiment 1, except that participants were required to overtly vocalize the phoneme at the sound-time (as opposed to covertly vocalize the phoneme, as in Experiment 1). If the observed increase in neural activity in the inner speech condition does in fact reflect motor preparation, a similar increase should also be observed during the preparatory period of overt speech.

## Experiment 2

### Materials and methods

#### Participants

Thirty-three participants enrolled in courses at UNSW Sydney were recruited in exchange for course credit or monetary payment. Three participants were excluded from the analysis for generating <50% of usable epochs in one or more conditions. The final sample consisted of 30 participants, of whom 22 were female, with a mean age of 26.23 (*SD* = 6.38) years.

#### Apparatus, stimuli, and procedure

The apparatus, stimuli, and procedure were identical to Experiment 1 (see Fig. 1), except that participants were instructed to overtly vocalize the phonemes (i.e. /BA/ or /BI/) at the sound-time—the *Active* condition. At the same moment, an audible phoneme (i.e. /BA/ or /BI/) was delivered to the participants’ headphones; this was produced by a male speaker, with a duration of 200 ms and loudness of 70 dB SPL. Each audible phoneme was presented on 50% of trials within each trial block. Participants were instructed to vocalize the overt phoneme softly to minimize the amount of bone conduction of the sound to the ears. Just as in Experiment 1, a *Passive* condition was also included. In addition, a *“Motor-Control”* condition in which participants overtly vocalized the phonemes but without any audible input at the sound-time was included. The purpose of this condition was to identify and correct for motor-related electrophysiological activity for analysis of poststimulus ERPs, and thus data from this condition were not included in the current analysis. For the poststimulus ERPs results, see Whitford et al. (2017).

#### E‌EG acquisition

The EEG acquisition was identical to Experiment 1.

#### E‌EG processing and analysis

The EEG processing and analysis were identical to Experiment 1. There was an average of 222.77 (*SD* = 13.06) usable epochs in the *Active* condition (of a max. possible 240 epochs), and 114.63 (*SD* = 5.08) in the *Passive* condition (of a max. possible 120 epochs). As in Experiment 1, the dependent variable was the mean amplitude of the late CNV in the 500 ms time-window preceding stimulus onset.

#### Statistical analysis

The statistical analysis was identical to Experiment 1.

### Results

**Fig. 3:**
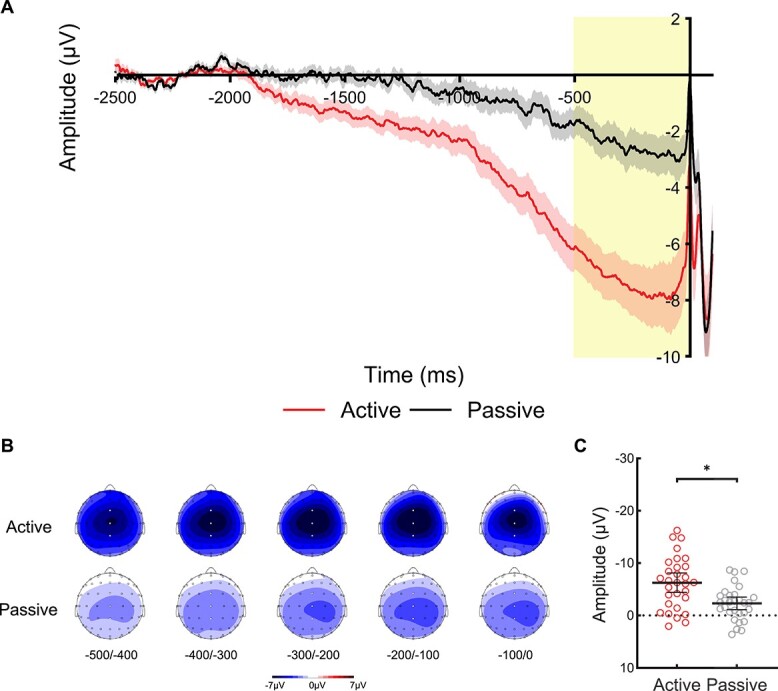
**CNV analysis of Experiment 2. (A)** Waveforms show the ERPs during the prestimulus period at electrode Cz in the active condition (red/bottom line) and passive condition (black/top line), and the shaded area represents the standard error of the mean. The yellow bar shows the late CNV time-window (−500 to 0 ms). The waveforms are shown collapsed across trials with audible phoneme /BA/ and /BI/ at stimulus onset (0 ms), and the waveform for the active condition is shown collapsed across inner phoneme /BA/ and /BI/ during the overt speech task. **(B)** Voltage maps are plotted separately for each condition showing the distribution of voltages over the scalp during the late CNV time-window for successive 100 ms time-windows from −500 ms until stimulus onset at 0 ms; white dots illustrate the electrodes FCz, Cz, and CPz used in the analysis. **(C)** Scatterplots show the mean amplitude of the late CNV time-window for the active and passive conditions averaged across electrodes FCz, Cz, and CPz. Each point represents one participant’s mean amplitude averaged across trials. These individual mean amplitudes were approximately normally distributed with no clear outliers. The horizontal bars represent the mean, and the error bars represent the 95% confidence interval. A significant contrast (*P* < 0.05) is indicated with an asterisk.

Figure 3A shows the ERPs during the prestimulus period at electrode Cz. Figure 3B shows the voltage maps during the late CNV time-window for successive 100 ms time-windows from 500 ms before stimulus onset. Figure 3C shows a scatterplot of the mean amplitude of the late CNV time-window averaged across electrodes FCz, Cz, and CPz for each condition. These individual mean amplitudes were approximately normally distributed with no clear outliers. One-sample *t*-tests revealed that the late CNV amplitude was significantly greater than the baseline in both the *Active* condition, *t*(29) = 6.92, *P* < 0.001, *d* = 1.26, 95% CI = (−8.130, −4.422), and in the *Passive* condition, *t*(29) = 4.03, *P* < 0.001, *d* = 0.74, 95% CI = (−3.539, −1.156). This indicates that a late CNV waveform was elicited in both conditions, which is maximal over the medial region and has a bilateral topographic distribution (see Fig. 3B, voltage maps). Moreover, a paired-sample *t*-test found that the late CNV amplitude in the *Active* condition was significantly larger than the *Passive* condition, *t*(40) = 3.55, *P* = 0.001, *d* = 0.65, 95% CI = (−6.193, −1.663). This indicates that the production of overt speech generated a significantly more negative late CNV than passive listening. Overall, Experiment 2 revealed the same pattern of results as Experiment 1.

In Experiment 3, we explored the influence of stimulus predictability on the amplitude of the late CNV preceding inner speech. Influential models of motor control suggest that predictions about forthcoming sensory consequences of self-generated actions are generated prior to the execution of the movements to subsequently modulate perceptual processing of these expected sensory input (Wolpert et al. 1995; Friston 2005). Indeed, previous research has shown that the brain encodes expected sensory consequences of a motor action in preparatory neural activity, resulting in a larger RP amplitude for overt actions that are followed by predictable sensory effects, compared with when action-effects are absent (Reznik et al. 2018; Vercillo et al. 2018; Wen et al. 2018; Pinheiro et al. 2020b). To assess whether this effect extends to inner speech—an imagined action—we manipulated the conditional probability of the audible phoneme presented simultaneously at the sound-time. Specifically, one audible phoneme was twice as likely to be presented as the other audible phoneme (i.e. 66 vs. 33% probability), such that prior expectations of the identity of the audible phoneme could be formed before the action-effect onset. We compared the amplitude of the late CNVs to predictable stimuli in Experiment 3 with the late CNVs in Experiment 1, in which both audible phonemes were equally likely to occur (i.e. 50 vs. 50% probability) so that the identity of the stimuli was unpredictable.

## Experiment 3

### Materials and methods

#### Participants

Fifty participants enrolled in a first-year Psychology course at UNSW Sydney were recruited in exchange for course credit. One participant was excluded from the analysis due to excessive artifacts in the EEG recording. One was further excluded for self-report diagnosis of a psychiatric disorder. The final sample consisted of 48 participants, of whom 33 were female, with a mean age of 19.88 (*SD* = 2.18) years.

#### Apparatus, stimuli, and procedure

Minor modifications were made to the experimental setup. First, the pair of headphones was replaced with a Sennheiser HD201. Second, the ticker tape in the countdown animation was replaced with a thicker white horizontal line. Third, the experiment consisted of 12 trial blocks, with 30 trials in each block. The rest of the apparatus, stimuli, and procedure were identical to Experiment 1. Specifically, the tasks participants were asked to perform were the same for the *Active* and *Passive* conditions, respectively.

In addition, on a random half of the blocks, the audible phoneme /BA/ was presented on 66% of trials, while the audible phoneme /BI/ was presented on 33% of trials (i.e. high /BA/ probability blocks). On the other half of the blocks, the audible phoneme /BI/ was presented on 66% of trials, while the audible phoneme /BA/ was presented on 33% of trials (i.e. high /BI/ probability blocks). Crucially, participants were informed of the stimulus probability at the beginning of each block, such that they were aware of the likelihood of hearing a particular audible phoneme within a trial block. In other words, the content of the audible phoneme was expected prior to stimulus onset. It is important to note that this manipulation of predictability was distinct from the congruency in the content of the inner and audible phonemes. For example, it was possible for a participant to expect to hear an incongruent phoneme (e.g. when producing the inner phoneme /BI/ in an environment in which 66% of the audible phonemes were /BA/). The order of the trial blocks was randomized.

#### E‌EG acquisition

The EEG acquisition was identical to Experiment 1.

#### E‌EG processing and analysis

The EEG processing and analysis were identical to Experiment 1. There was an average of 222.58 (*SD* = 17.06) usable epochs in the *Active* condition (of a max. possible 240 epochs), and 108.98 (*SD* = 12.40) in the *Passive* condition (of a max. possible 120 epochs). Similar to Experiment 1, the dependent variable was the mean amplitude of the late CNV in the 500 ms time-window preceding stimulus onset.

#### Statistical analysis

First, to replicate the results of Experiment 1, one-sample *t*-tests (two-tailed) comparing the late CNV amplitude to zero were performed for the *Active* and *Passive* conditions, respectively, to establish the existence of a late CNV waveform in each condition. This was followed by a paired-sample *t*-test (two-tailed) to compare the amplitude of the late CNV between the *Active* and *Passive* conditions. In addition, to examine the effect of stimulus predictability, a 2 × 2 mixed ANOVA with within-subjects factor *Condition* (*Active* and *Passive*) and between-subjects factor *Probability* (*50* and *66%*) was tested to compare the amplitude of the late CNV in both conditions across Experiment 1 (unpredictable stimuli) and Experiment 3 (predictable stimuli). In the case of a main effect or interaction, contrasts were used to unpack the simple effects. Welch’s *t*-test was used in the case of unequal variances (Delacre et al. 2017), and the Greenhouse–Geisser correction was used in the case of a violation in the assumption of sphericity. All analyses were conducted using electrode Cz as it has been shown to be maximal in both conditions, and to be consistent with previous studies (Vercillo et al. 2018; Wen et al. 2018; Pinheiro et al. 2020b).

### Results

**Fig. 4:**
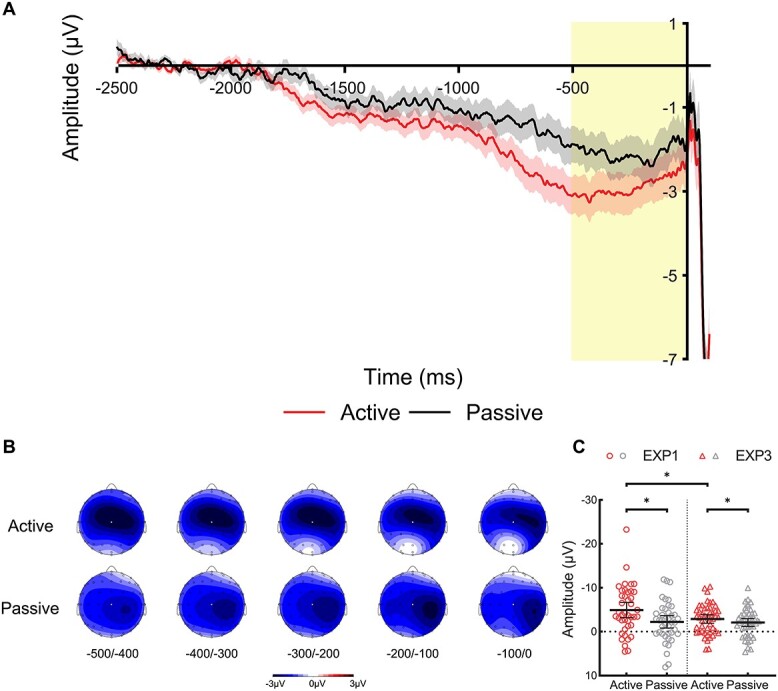
**CNV analysis of Experiment 3. (A)** Waveforms show the ERPs during the prestimulus period at electrode Cz in the active condition (red/bottom line) and passive condition (black/top line), where prior expectations of the identity of the upcoming audible phoneme could be formed with probablistic manipulations. The shaded area represents the standard error of the mean. The yellow bar shows the late CNV time-window (−500 to 0 ms). The waveforms are shown collapsed across trials with audible phoneme /BA/ and /BI/ at stimulus onset (0 ms), and the waveform for the active condition is shown collapsed across inner phoneme /BA/ and /BI/ during the inner speech task. **(B)** Voltage maps are plotted separately for each condition showing the distribution of voltages over the scalp during the late CNV time-window for successive 100 ms time-windows from −500 ms until stimulus onset at 0 ms; the white dot illustrates the electrode Cz used in the analysis. **(C)** Scatterplots show the mean amplitude of the late CNV time-window for the active and passive conditions at electrode Cz in Experiment 1 (unpredictable stimuli; circles) and Experiment 3 (predictable stimuli; triangles). Each point represents one participant’s mean amplitude averaged across trials. These individual mean amplitudes were approximately normally distributed with no clear outliers. The horizontal bars represent the mean, and the error bars represent the 95% confidence interval. Significant contrasts (*P* < 0.05) are indicated with asterisks.

Figure 4A shows the ERPs during the prestimulus period at electrode Cz. Figure 4B shows the voltage maps during the late CNV time-window for successive 100 ms time-windows from 500 ms before stimulus onset. Figure 4C shows a scatterplot of the mean amplitude of the late CNV time-window for the Active and Passive conditions at electrode Cz in Experiment 1 (unpredictable stimuli) and Experiment 3 (predictable stimuli). These individual mean amplitudes were approximately normally distributed with no clear outliers. One-sample *t*-tests revealed that, in Experiment 3, the late CNV amplitude was significantly greater than the baseline in both the *Active* condition, *t*(47) = 6.02, *P* < 0.001, *d* = 0.87, 95% CI = (−3.851, −1.922), and in the *Passive* condition, *t*(47) = 4.64, *P* < 0.001, *d* = 0.67, 95% CI = (−2.993, −1.182). Moreover, a paired-sample *t*-test found that the late CNV amplitude in the *Active* condition was significantly larger than the *Passive* condition, *t*(47) = 2.04, *P* = 0.047, *d* = 0.30, 95% CI = (−1.586, −0.127). These findings mirror the general pattern that was observed in Experiment 1.

Furthermore, a mixed ANOVA revealed a significant main effect of *Condition*, *F*(1,87) = 20.07, *P* < 0.001, η_p_^2^ = 0.19, and a significant interaction between *Condition* and *Probability*, *F*(1,87) = 5.87, *P* = 0.018, η_p_^2^ = 0.06. However, there was no significant main effect of *Probability*, *F*(1,87) = 1.92, *P* = 0.170, η_p_^2^ = 0.02. Follow-up contrasts found that the late CNV in the *Active* condition was significantly more negative under 50% stimulus probability (Experiment 1) than 66% stimulus probability (Experiment 3), *t*(63.67) = 2.07, *P* = 0.042, *d* = 0.46, 95% CI = (−4.000, −0.073). However, no significant difference was found between the *Passive* conditions, *t*(87) = 0.19, *P* = 0.850, *d* = 0.04, 95% CI = (−1.762, 1.455). These results indicate that stimulus predictability has a specific modulatory effect on inner speech preparation and action-effect contingency more generally, which could not be accounted for by mere expectation of stimulus identity without the involvement of motor action (i.e. passive listening). An exploratory analysis also revealed no significant difference in the late CNV in Experiment 3 between expecting an action-congruent outcome (e.g. imagine /BA/ in high /BA/ probability blocks) or action-incongruent outcome (e.g. imagine /BA/ in high /BI/ probability blocks), *t*(47) = 0.49, *P* = 0.629, *d* = 0.07, 95% CI = (−0.513, 0.840).

## Discussion

The present study investigated whether the production of inner speech would be preceded by a prestimulus activity such as has traditionally been associated with the execution of overt motor actions. Using a cue paradigm in which participants were asked to imagine an inner phoneme or to passively listen to an audible phoneme at a cued moment, we observed a significantly more negative slow drift than baseline in both conditions, which we denoted as a late CNV. The fact that a late CNV was elicited when no (overt) motor response was required is consistent with previous findings demonstrating that a perceptual task alone is sufficient to generate a late CNV (Simons et al. 1979; Ruchkin et al. 1986), and supports the notion that the late CNV indexes nonmotoric components such as stimulus anticipation (Brunia and Damen 1988; Cui et al. 2000; Mento 2013). Critically, as hypothesized, the late CNV in the inner speech condition was significantly more negative than the Passive condition. This result suggests that the preparation to produce inner speech generates a late CNV similar to that observed in the preparation of overt speech (Michalewski et al. 1977; Vanhoutte et al. 2015), which reflects motor preparation beyond nonmotoric contributions. This result extends previous findings of a motoric CNV (Beisteiner et al. 1995; Cunnington et al. 1996, 1997; Jankelowitz and Colebatch 2002) and RP (do Nascimento et al. 2006; Niazi et al. 2013; Park et al. 2022) preceding the onset of motor imagery, and demonstrates that a motor-related slow wave also precedes the onset of inner speech in the absence of any overt motor action.

We replicated the finding of a larger late CNV amplitude in the inner speech condition than the Passive condition (Experiment 3), and further demonstrated an effect of stimulus predictability on the amplitude of the late CNV preceding inner speech. Specifically, we observed a smaller late CNV in the inner speech condition when expectations as to the identity of the audible phoneme could be formed, compared with the situation in which no such expectations could be formed. However, no such difference was observed between the two Passive conditions. These results suggest that the mere expectation of stimulus identity without any motor action does not modulate the late CNV and is consistent with previous research that found no RP preceding expectations of the presentation of auditory stimuli (Reznik et al. 2018). The fact that the late CNV of inner speech is sensitive to the expectations of sensory outcomes aligns with the idea of an internal forward model that uses predictions to modulate the processing of sensory consequences of voluntary actions (Wolpert et al. 1995; Wolpert and Miall 1996) and supports the view that inner speech may be conceived as “a kind of action” (Feinberg 1978).

Contrary to previous research that found an enhanced RP preceding actions that were followed by predictable outcomes (Reznik et al. 2018; Vercillo et al. 2018; Wen et al. 2018; Pinheiro et al. 2020b), we found that the late CNV was smaller preceding inner speech when the identity of the audible phoneme was predictable by comparing the results of Experiments 1 and 3. To our knowledge, this effect has not been reported previously. One key difference between our study and previous research is that in our design, the “action” (i.e. producing an inner phoneme) and the related “effect” (i.e. an audible phoneme) had a 100% contingency, which was operationalized by prompting participants to produce an inner phoneme while simultaneously presenting an audible phoneme. Only the expectations of stimulus identity were manipulated. In contrast, previous studies have manipulated the level of action-effect contingency, such that conditions with 100% contingency were compared with conditions where an action could trigger a stimulus 50% of the time (Wen et al. 2018), or 0% of the time (Reznik et al. 2018; Vercillo et al. 2018; Pinheiro et al. 2020b). Thus, we reason that the late CNVs preceding inner speech in Experiments 1 and 3 both encoded the expected sensory consequences of (covert) actions as previously suggested (e.g. I may hear an audible phoneme /BA/ when I produce an inner phoneme /BA/) (Ford et al. 2014), but having an expectation of the stimulus identity in Experiment 3 allowed for a more *precise* action-effect prediction to be made (e.g. I will hear an audible phoneme /BA/ when I produce an inner phoneme /BA/). This idea fits well with broader computational models that emphasize the role of precision estimation of our predictions in generating reliable inferences regarding the sensory environment (Friston 2005, 2017; Press et al. 2020; Yon and Frith 2021), and suggests a case where the probabilistic structure of stimuli in the environment, and more specifically, the expectations and degree of certainty regarding the fulfillment of sensorimotor predictions (i.e. from ambiguous to likely a match or mismatch in speech content), modified the amplitude of the late CNV. This effect may be unique to the motor system given the fact that the passive CNV was unaffected, and is corroborated by Voss et al. (2008) which found that probabilistic manipulation of which hand (i.e. left or right hand) would be cued led to premovement sensory attenuation of the more probable hand.

Interestingly, this reduction in neural activity preceding predictable action-effects is consistent with a recent study, which found a decrease of EEG power specific to the flickering frequencies (i.e. 6 or 10 Hz) of predictable visual action-effects before their presentation (Dignath et al. 2020), and the broader findings of a decrease in oscillatory activity during action preparation (e.g. Pogosyan et al. 2009). Collectively, these findings provide evidence for the representation of *specific* action-effect predictions during the preparation of actions, and that they may begin to suppress the activity of sensory units in anticipation of the predicted stimuli (Voss et al. 2008)—a view in concordance with computational models of action control (Wolpert and Miall 1996; Wolpert et al. 2011).

Notably, a recent study by Li et al. (2020) demonstrated the precision of forward model predictions during different “speech preparation” phases with an alternative approach. In a delayed-articulation task, participants were prompted by a meaningless visual cue to prepare to speak in a few seconds (i.e. general preparation), or by a written syllable, which informed them of the specific phoneme to be spoken (i.e. specific preparation) (Li et al. 2020). During the preparatory stage (i.e. before overt articulation), an audible phoneme was presented. Intriguingly, general preparation resulted in a suppression of the auditory N1 elicited by the auditory probe, whereas specific preparation led to an enhancement of N1 only in response to the prepared phoneme, compared with a passive listening condition. These results suggest that the specificity of speech preparation, and more importantly, the precision of action-effect predictions made during preparation, could modulate auditory neural responses in different ways. On the face of it, the enhanced sensory processing to predicted sensations in Li et al. (2020) seems to be at odd with the functional role of sensory predictions stipulated by traditional “cancellation” theories (Wolpert et al. 1995; Blakemore et al. 1998). Indeed, an emerging body of work that has demonstrated a perceptual enhancement effect on expected sensory outcomes has prompted a debate of the nature and adaptive functions of predictions—see Reznik and Mukamel (2019), Press et al. (2020), and Press et al. (2023) for a more detailed discussion. How the availability and unfolding of motor signals during the preparatory stage relate to the sensory processing of stimuli is still unclear and warrants further investigation.

Overt actions are almost always accompanied by corresponding sensations. Previous studies that have attempted to isolate efference copy-based predictions embedded within preparatory motor signals have generally done so by looking at their modulatory effect on external stimulation before action execution by, for instance, using transcranial magnetic stimulation to delay their dispatch to later stages of the motor hierarchy (Voss et al. 2006), or employing prediction-specific probes (Fuehrer et al. 2022). Inner speech, which has been suggested to share the same underlying neural underpinnings as overt speech (Tian and Poeppel 2010, 2012, 2013; Alderson-Day and Fernyhough 2015) but without any sensory consequences, may thus provide a novel avenue to examine sensorimotor predictions during preparation. Specifically, in the context of the present paradigm, it is conceivable to include a condition in which inner speech is produced without any auditory feedback to determine whether the amplitude of the late CNV would be further reduced in the absence of any sensory consequences.

Of course, our mental actions do not ordinarily have sensory consequences. By artificially creating a situation in which they do, it is possible that the modulation on the CNV amplitude in the present study was a result of the learned contingency between an imagined action and an external stimulus (i.e. similar to learning that pressing a button will generate a tone), as well as the experience of agency (see Jo et al. 2014; Han et al. 2021, 2022), such that inner speech in everyday life may not typically recruit a motor-based prediction mechanism. However, the fact that the production of inner speech attenuated auditory stimuli in a content- and temporal-specific manner without any prior learning (Whitford et al. 2017; Jack et al. 2019), which are characteristic features of the forward models (for how overt and covert speech may rely on a shared mechanism of feedforward prediction, see Niziolek et al. 2013; also see Whitford et al. 2017), provide evidence in favor of the tenet that inner speech engages the same (or substantially overlapping) neural mechanism as overt speech at various stages of the motor hierarchy, including motor preparation (see Kilteni et al. 2018 for an example of motor imagery).

To provide a point of reference to Experiment 1, in Experiment 2 we asked participants to overtly (as opposed to covertly) vocalize the phoneme. The same pattern of results was revealed: A late CNV was elicited in both the overt speech and Passive conditions, with a more negative amplitude preceding overt speech. Interestingly, the late CNV amplitude preceding overt speech production was seen to be much larger than the late CNV preceding inner speech production (compare scales in Figs. 2A and 3A). This finding is consistent with previous studies, which found that overt motor execution generated a larger slow wave amplitude than motor imagery (Cunnington et al. 1996; Park et al. 2022). It also aligns with brain imaging studies that reported reduced activation in overlapping brain regions during inner speech relative to overt speech (for a review, see Perrone-Bertolotti et al. 2014), with several studies showing little to no contribution from the primary motor cortex during covert articulation (Palmer et al. 2001; Shuster and Lemieux 2005; Pei et al. 2011). The bilateral topographic distribution over the midline (see voltage maps) in both the inner speech and overt speech conditions aligns with previous findings in simple speech production (Michalewski et al. 1977; McArdle et al. 2009), which may reflect contributions from the pre-SMA and SMA (Picard and Strick 1996; Nachev et al. 2008): regions that are also implicated in auditory processing and auditory imagery (see Lima et al. 2016 for a review). Taken together, the present study provides the strongest evidence to date that inner speech is associated with a motoric late CNV, which reflects motor preparatory processes similar to overt actions.

It is important to note the presence of several event-preceding negativities, such as the CNV and RP, as well as the stimulus-preceding negativity (SPN). The SPN is a nonmotor anticipatory slow potential that occurs prior to stimuli that carry significant information (van Boxtel and Böcker 2004). The resemblance in their morphology, topography, and latency has complicated the understanding of the relationships among these slow waves (Schurger et al. 2021). Some have ascertained that the late CNV is identical to the RP (Rohrbaugh et al. 1976; Gaillard 1977). However, studies with patients with lesion or neurodegenerative disorders indicated different neural substrates between the two (Ikeda et al. 1994, 1997). The comparison is further muddled by the complexity of the motor task (Cui et al. 2000). It is also possible that the CNV is a composite of the RP and SPN (Damen and Brunia 1987; van Boxtel and Brunia 1994b; Kotani et al. 2011), though the summation of these components did not result in a scalp distribution that perfectly mirrored that of the CNV (Kotani et al. 2011; Brunia et al. 2012). While we called the slow cortical potentials the CNV in the present study because the (mental) actions were stimulus-driven, we were open to the possibility of it being something else, like the RP. We look forward to distinguishing between these negative slow waves in the future.

An alternative explanation for the results of Experiment 1 could be that attentional differences between the conditions may have influenced the results. However, the observation of a significant late CNV even during passive listening suggests the involvement of attention, which has been regarded as necessary for CNV generation (Walter et al. 1964; Tecce 1972). Excluding trials with low ratings of performance also helped ensure, to some extent, that participants were actively engaged in each condition, although we concede that the level of attention may have varied between them. It is also worth noting that previous research found that RP preceding finger movements was not affected by selective attention, but instead was reduced with increasing cognitive control and working memory demands (Baker et al. 2011). Thus, it is improbable that these factors account for the larger amplitude of the late CNV observed in the inner speech condition. Another possibility is that the enhanced negativity observed in the inner speech condition may not be due to inner speech production per se, but rather may reflect some form of mental effort, which has been shown to elicit a CNV without an overt response (e.g. mental counting or judging a time-interval) (Walter et al. 1964; Bares et al. 2007). A recent study also indicated that a mental decision without a motor response is sufficient to evoke an RP (Alexander et al. 2016). While it is acknowledged that determining the specificity of inner speech’s contribution to prestimulus activity in the current design is not feasible, analysis of the poststimulus ERPs of the current data in Whitford et al. (2017) provides strong evidence that inner speech, similar to overt speech, is associated with a content-specific and temporally precise efference copy. As such, we conclude that the late CNV in the inner speech condition likely reflects motor preparatory activity.

Establishing the existence of preparatory motor activity to inner speech holds important implications for understanding some of the characteristic symptoms associated with schizophrenia, such as auditory verbal hallucinations. An influential account suggests that auditory verbal hallucinations may arise as a result of misattribution of one’s inner speech to an external source (Feinberg 1978; Frith 1987). These deficits may already be present during motor planning in patients with schizophrenia (Ford et al. 2014; Pinheiro et al. 2020a). Converging evidence also suggests blunting of CNV amplitude in patients with schizophrenia (for a review, see Osborne et al. 2020). However, it is currently not known whether patients show deficits in inner speech preparation as indexed by CNV. The current protocol may thus provide a useful framework for directly testing these long-held but hitherto untestable ideas in the future.

## Conclusions

The present study demonstrated that the production of inner speech is preceded by a motor-related neurophysiological signature. Specifically, we found that producing an inner phoneme elicited a motoric late CNV that cannot be attributed to nonmotoric anticipatory attention, and which differed in amplitude from a passive listening condition (Experiment 1). These results suggest that inner speech is associated with motor preparatory activity typically observed in overt movement, and extend upon a growing body of literature that shows that mental actions are sufficient to generate a premovement negative slow wave (Park et al. 2022). These findings were corroborated by an identical experiment that engaged in overt speech, which revealed the same pattern of results (Experiment 2). Furthermore, the late CNV to inner speech was modulated by the predictability of action-effect, suggesting that specific sensory predictions may be generated prior to covert action and encoded in the neural activity during motor preparation (Experiment 3), as has previously been demonstrated for overt actions (Reznik et al. 2018; Vercillo et al. 2018; Wen et al. 2018; Pinheiro et al. 2020b). Collectively, these findings imply that inner speech may be conceived as “a kind of action” that is similar to overt speech, and may ultimately represent our most complex motor act.

## Data Availability

Data for this study can be found at https://osf.io/gfn6b/.

## Notes

*Conflict of interest statement*: A.W.F.H. has received consultancy fees from Janssen Australia, Lundbeck Australia and Seqirus. He has received payments for educational sessions run for Lundbeck Australia and Servier. He has developed educational material for Servier. He is the recipient of an investigator initiated grant from the Balnaves Foundation and Takeda Pharmaceutical Company. He is an investigator on an industry sponsored trial by Alto Neuroscience. He is the recipient of funding from the Australian Research Council, the Medical Research Futures Fund, and the National Health and Medical Research Council. He has received philanthropic funding from The Balnaves Foundation. He is the chair of One Door Mental Health. The other authors have no competing interests to declare.
